# Robotic-assisted thoracic surgery in children with congenital lung disease: advancements, challenges, and future directions

**DOI:** 10.3389/fped.2026.1822207

**Published:** 2026-05-15

**Authors:** Masato Kojima

**Affiliations:** Department of Pediatric Surgery, Hiroshima University Hospital, Hiroshima, Japan

**Keywords:** congenital lung disease, minimally invasive surgery, pediatric thoracic surgery, pediatrics, robotic-assisted thoracic surgery

## Abstract

**Background:**

Congenital lung disease (CLD), including congenital pulmonary airway malformation and pulmonary sequestration, is increasingly diagnosed with advances in prenatal and postnatal imaging. Video-assisted thoracoscopic surgery (VATS) is widely accepted as the standard minimally invasive approach. Robotic-assisted thoracic surgery (RATS) has emerged as an alternative platform, but its role in pediatric pulmonary resection remains to be defined.

**Methods:**

A narrative review was conducted using PubMed from database inception to February 2026. Search terms included “robotic-assisted thoracic surgery,” “pediatric,” and specific congenital lung anomalies. Clinical studies, comparative cohorts, and case series reporting RATS for pediatric CLD were included. Findings were qualitatively synthesized.

**Results:**

Three studies were included, all of which were comparative cohorts. Robotic resections were mainly performed for congenital pulmonary airway malformation and pulmonary sequestration. The youngest reported patient was 6 months old (8 kg). Conversion rates were low (1%–3%), and major intraoperative complications were uncommon. Postoperative recovery was acceptable, with short hospital stays and predominantly minor complications. Comparative analyses showed similar perioperative safety profiles between RATS and VATS, although operative times were generally longer with RATS. Reported advantages of RATS included enhanced three-dimensional visualization, improved instrument articulation, and ergonomic benefits.

**Conclusions:**

RATS is a feasible and safe option for selected pediatric patients with CLD. While short-term outcomes are comparable to VATS, clear superiority has not been demonstrated. Prospective multicenter studies and long-term functional evaluation are required to define its role in pediatric thoracic surgery.

## 1 Introduction

Congenital lung disease comprises a heterogeneous spectrum of developmental pulmonary anomalies, including congenital pulmonary airway malformation (CPAM), bronchopulmonary sequestration, congenital lobar overinflation, and bronchogenic cyst (1). Advances in prenatal and postnatal imaging have led to increased detection of these conditions, facilitating early diagnosis and surgical planning (2). While some congenital pulmonary lesions may remain asymptomatic, the natural history of these abnormalities is not always benign; recurrent respiratory infections, pneumothorax, progressive hyperinflation, and potential malignant transformation have been described, supporting surgical management in selected patients even in the absence of symptoms (1).

Over the past two decades, pediatric thoracic surgery has undergone substantial transformation driven by minimally invasive approaches. Video-assisted thoracoscopic surgery (VATS) has become widely adopted for congenital lung disease, and thoracoscopic lobectomy is increasingly regarded as the preferred procedure. Growing clinical evidence supports VATS as an effective technique with favorable perioperative outcomes compared with open thoracotomy, including reduced postoperative pain, improved cosmesis and better pulmonary function (3–5).

Nevertheless, thoracoscopic pulmonary resection in children remains technically challenging. Pediatric patients present unique anatomic constraints—small thoracic cavities, narrow intercostal spaces, and fragile hilar structures, making precise vascular and bronchial dissection difficult. These challenges are accentuated in infants and small children, in whom the operative field is limited, and instrument crowding is common. Moreover, pediatric thoracoscopic lobectomy requires significant surgical experience to achieve consistent and reproducible outcomes (6–8). Robotic-assisted thoracic surgery (RATS) has emerged as a promising alternative platform for complex thoracic procedures. Robotic systems offer three-dimensional high-definition visualization, tremor filtration, and wristed instruments with increased degrees of freedom that improve triangulation and enable fine dissection and suturing within confined spaces (9, 10). In adult thoracic surgery, RATS has expanded rapidly, and its advantages in complex hilar work and lymph node dissection have driven broader adoption. In pediatric surgery, however, the implementation of robotic approaches has progressed more cautiously due to a distinct set of limitations, including patient size, trocar spacing constraints, external arm collision, and the lack of appropriately miniaturized instruments for neonates and small infants (11–13).

In recent years, pediatric thoracic robotic literature has matured from isolated feasibility reports to broader synthesis efforts. Notably, a 2025 systematic review and meta-analysis focusing specifically on RATS in children highlighted that RATS can be considered safe and effective for selected pediatric thoracic conditions, while simultaneously underscoring that available evidence remains limited by small sample sizes, retrospective design, and institutional heterogeneity (14).

Given the increasing interest in robotics and the expanding technical capabilities of robotic platforms, clarifying the present and future role of RATS in pediatric congenital lung disease is timely (14). A comprehensive review is warranted not only to summarize the available clinical outcomes but also to examine the underlying reasons why robotics may offer unique value for pediatric pulmonary resection.

Therefore, this review aims to (1) synthesize current evidence on robotic-assisted thoracic surgery for congenital lung disease in children, (2) analyze technical and perioperative challenges specific to the pediatric population, and (3) outline future directions.

## 2 Methods

### 2.1 Literature search

A comprehensive narrative review of the literature was conducted to identify studies evaluating RATS in pediatric patients with congenital lung disease. Electronic searches were performed in PubMed from database inception to February 2026.

Search terms included combinations of:

“robotic-assisted thoracic surgery”, “robotic surgery”, “RATS”,

“pediatric”, “children”, “infant”,

“congenital lung disease”, “congenital pulmonary airway malformation”, “CPAM”, “pulmonary sequestration” and “congenital lobar overinflation”.

Reference lists of included studies and relevant review articles, and editorials were manually screened to identify additional eligible studies.

### 2.2 Study selection and scope

Original clinical studies, comparative analyses, institutional case series, and pertinent review articles reporting the use of RATS for congenital lung disease in children were included. Given the evolving nature of pediatric robotic thoracic surgery and the limited number of large-scale studies, smaller series and early feasibility reports were also considered to provide a comprehensive overview of current experience. Studies focusing exclusively on adult populations were excluded. In addition, non-English language articles, conference abstracts without available full text, editorials, and reports lacking sufficient clinical or perioperative outcome data were excluded.

### 2.3 Data extraction and synthesis

Key data were extracted regarding patient demographics, type of congenital lung lesion, surgical approach, technical feasibility, perioperative outcomes, and postoperative recovery. Given the heterogeneity of study designs and reported outcome measures, findings were synthesized qualitatively to highlight trends, technical considerations, and evolving indications for RATS in pediatric thoracic surgery.

### 2.4 Methodological considerations

**Figure 1:**
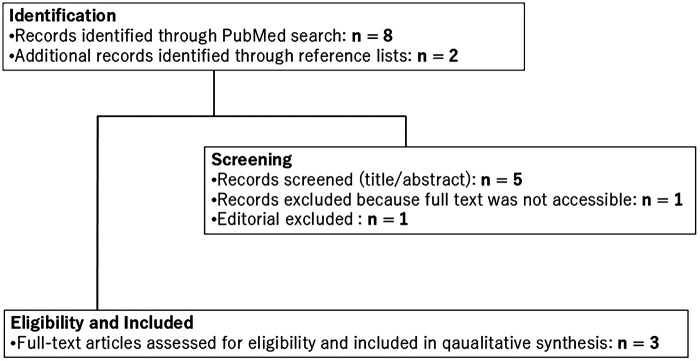
Study selection flow diagram.

This narrative approach aimed to integrate available evidence while acknowledging inherent limitations, including the use of a single database (PubMed), retrospective study designs, and small patient cohorts. To enhance transparency and reproducibility, the study selection process has been clearly illustrated in a flow diagram (Figure 1). Emphasis was placed on identifying technical advancements, reported benefits, and ongoing challenges associated with robotic pulmonary resection in children.

## 3 Results

### 3.1 Overview of included studies

A total of three studies were included in this narrative review, all of which were comparative retrospective cohort studies. All included studies were published within the past decade, reflecting the relatively recent adoption of RATS in pediatric populations. Sample sizes were generally small, consistent with the low incidence of congenital lung disease and the limited availability of robotic platforms in pediatric centers. All reports originated from high-volume tertiary institutions with established minimally invasive surgery programs (Table 1).

**Table 1:** The summary of included studies.

Case	Author	Year	Disease	Procedure	*N* (RATS)	*N* (VATS)	Age (median, range)	Weight (median, range)	Conversion	Operation time (median, range)	Complication	Postoperative hospital stay (median, range)	Robot
1	Li et al. (15)	2022	CPAM and IPS	Segmental resection, lobectomy	29	42	81 m, 6–156 m vs. 72 m, 2–149 m	25 kg, 8–81 kg vs. 23 kg, 6–56 kg	1 (3.4%) vs. 2 (4.7%)	148.3 min, 80–227 min vs. 118.3 min, 90–160 min	0 (0%) vs. 1 (2.3%)	3.5 days, 2–6 days vs. 3.8 days, 2–9 days	da Vinci Surgical Si System
2	Liang et al. (16)	2024	IPS	Sequestrectomy, segmental resection, lobectomy	93	77	10 m, 7–25 m vs. 9.5 m, 7–17.5 m	9.5 kg, 8.5–13.1 kg vs. 9.2 kg, 8.3–12 kg	1 (1.1%) vs. 1 (1.3%)	75 min, 60–92.5 min vs. 60 min, 40–70 min	30.1% vs. 20.8% (pneumothorax and pleural effusion)	5.0 days, 4–6 days vs. \6.0 days, 5–7 days	da Vinci Xi Robotic System
3	Wei et al. (17)	2024	CPAM	Segmental resection, lobectomy	94	94	12 m, 8–44.75 m vs. 12 m, 7–45 m	10 kg, 8.5–15 kg vs. 9.8 kg, 8.3–14.15 kg	0 (0%) vs. 0 (0%)	97.5 min, 79.0–116.5 min vs. 70 min, 50–90 min	28.7% vs. 24.5% (pneumonia, pneumothorax and pleural effusion)	5.0 days, 5–6 days vs. 6.0 days, 5–7 days	da Vinci Xi Robotic System

y, years; m, months; CPAM, congenital pulmonary airway malformation; IPS, intra pulmonary sequestration; RATS, robot-assisted thoracoscopic surgery; VATS, video-assisted thoracoscopic.

### 3.2 Patient characteristics and indications

All patients underwent robotic pulmonary resection for CPAM or pulmonary sequestration.

The included studies encompassed a broad age range, spanning from infancy to adolescence. Notably, the youngest reported patient undergoing RATS was 6 months old, with a minimum body weight of 8 kg (15). These findings suggest that robotic pulmonary resection has primarily been performed in patients with sufficient thoracic dimensions to accommodate port spacing and robotic arm movement. In a study, patient selection appeared to favor older infants and children, likely reflecting current technical and anatomical constraints (15).

### 3.3 Surgical procedures and technical feasibility

**Figure 2:**
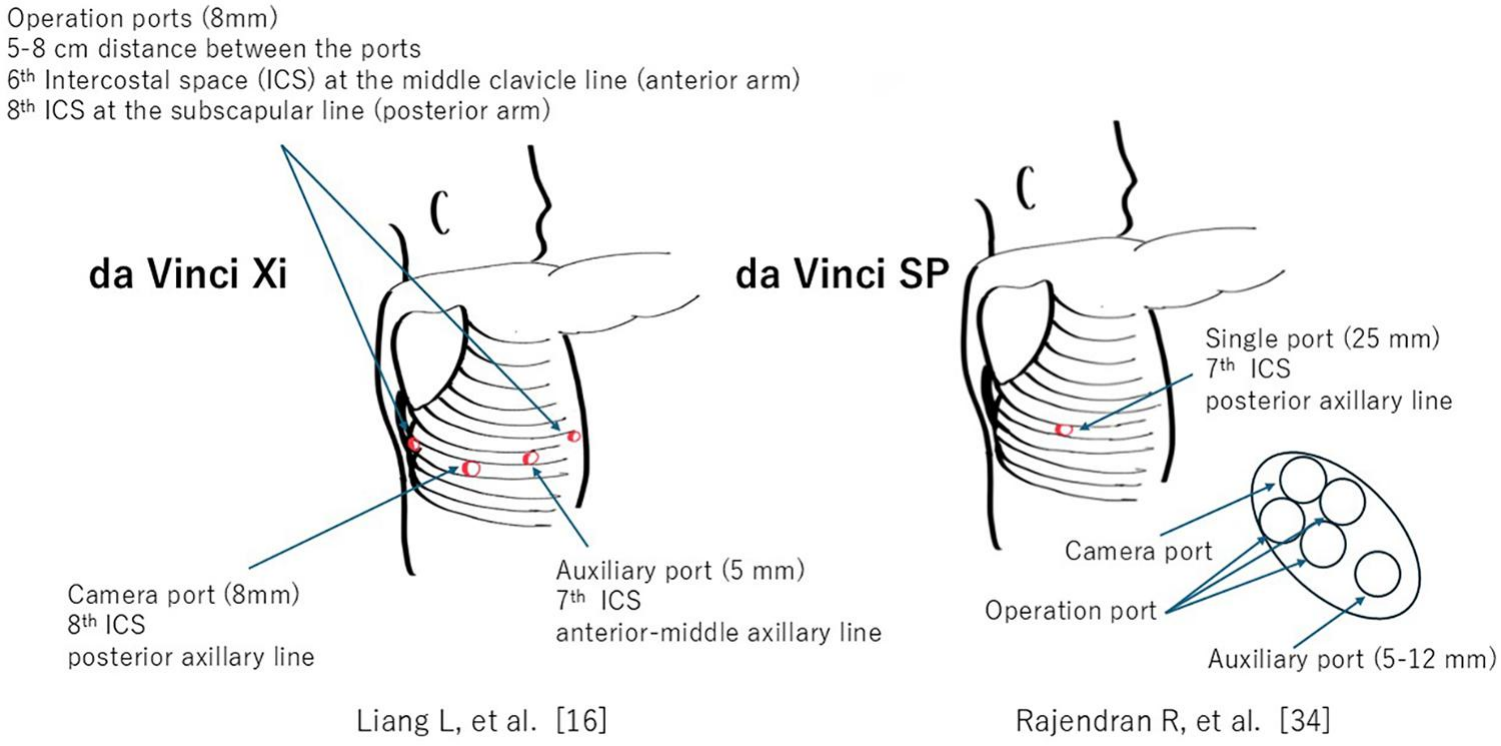
Representative port placement configurations for the da Vinci Xi and SP systems[Rajendran et al. (34)].

Robotic-assisted procedures included lobectomy and segmentectomy. Across studies, robotic pulmonary resection was technically feasible in carefully selected pediatric patients. Conversion rates to open surgery were low, reported at approximately 1.1% and 3.4% in comparative cohorts (15, 16). These findings suggest that, in experienced hands, RATS can be safely implemented with acceptable reliability. The differences including port placement among the surgical systems are shown in Table 2 and Figure 2.

**Table 2:** The advances of da Vinci robotic systems and advantages and limitations.

System	Advantage	Limitation
da Vinci S/Si (2000∼)	3D display	12-mm endoscope
	Seven degrees of freedom	Bulky arm design
	Motion scaling	Limited port flexibility
	Tremor filteration	Arm collision
da Vinci Xi (2014∼)	Overhead boom rotation	
	8-mm endoscope	
	Greater port placement flexibility	
	Slimmer arm architecture	
	Reduced arm clashing	
	Simple docking procedure	
da Vinci SP (2018∼)	Single port cannula	
	Articulating camera + multi-jointed instruments	

### 3.4 Perioperative outcomes

Operative times varied widely across institutions, ranging from approximately 60 to 227 min. This variability likely reflects differences in case complexity, surgeon experience, and institutional learning curves, as well as additional docking and setup time associated with robotic systems. Major intraoperative complications were uncommon. No procedure-related mortality was described.

### 3.5 Postoperative outcomes and recovery

The incidence of postoperative complications varied among studies, possibly reflecting differences in reporting standards and institutional practice patterns. Postoperative complication rates were reported as 30.1% and 28.7% in the two larger cohorts. Although these rates are not negligible, most complications were minor and managed conservatively. Reported adverse events included pneumonia, pneumothorax, and pleural effusion. No high-grade or life-threatening complications were documented.

Postoperative recovery was favorable across reports. The length of hospital stay was typically short, with most patients discharged around postoperative day five or earlier. Early chest tube removal and rapid return to baseline activity were commonly described, consistent with the minimally invasive nature of the procedure.

### 3.6 Comparison between RATS and VATS

All included studies were comparative cohort studies and evaluated RATS and VATS (15–17). These studies demonstrated comparable perioperative safety profiles between the two approaches, including similar complication and conversion rates, while operative times were consistently longer in the RATS group. Several authors also highlighted ergonomic benefits for the surgeon in RATS, suggesting reduced physical strain and improved operative comfort during prolonged procedures (15).

### 3.7 Long-term and functional outcomes

Data on long-term outcomes remain limited due to the relatively recent introduction of robotic thoracic surgery in pediatric practice. Available studies reported favorable short- to mid-term follow-up, with no evidence of recurrent disease or compromised pulmonary function development. The absence of large-scale prospective studies and standardized long-term follow-up protocols limits definitive conclusions regarding durability and functional superiority.

### 3.8 Summary of key findings

Overall, the available evidence suggests that RATS is a feasible and safe approach for selected pediatric patients with congenital lung disease. Robotic pulmonary resection has been successfully performed across a broad age range, including infants as young as 6 months and weighing as little as 8 kg. Although operative times were generally longer compared with conventional thoracoscopic approaches, perioperative safety profiles were comparable. Postoperative recovery was favorable, with minor complications and short hospital stays. However, long-term outcome data remains limited, underscoring the need for further prospective and multicenter studies.

## 4 Discussion

RATS for congenital lung disease in children represents an evolving frontier in minimally invasive pediatric surgery. While conventional thoracoscopic surgery has achieved broad acceptance as the standard minimally invasive approach, robotic platforms introduce distinct technological capabilities that may reshape the surgical management of complex congenital pulmonary lesions.

The principal theoretical advantages of robotic systems include three-dimensional magnified visualization, motion scaling, tremor filtration, and wristed instruments with seven degrees of freedom (18). These features are particularly relevant in pediatric pulmonary resection, where fine broncho vascular structures must be dissected within a confined thoracic cavity. Experimental and adult thoracic studies have demonstrated that robotic articulation enhances precision during hilar dissection and intracorporeal suturing (19, 20).

In children, the small caliber of pulmonary arteries and bronchi increases the margin for technical errors. Enhanced dexterity and stable visualization may therefore translate into safer vessel isolation and bronchial division, especially during segmentectomy or anatomically complex resections. Cadaveric and simulation-based analyses suggest that robotic systems may improve instrument triangulation in narrow operative fields compared with rigid thoracoscopic instruments (21, 22).

Although recent institutional series have reported generally acceptable outcomes, large prospective pediatric datasets remain limited. Several contemporary single-center experiences have described low intraoperative complication rates and acceptable conversion rates during robotic lobectomy and segmentectomy in children (15–17). However, reported postoperative complication rates of approximately 30% in larger cohorts indicate that adverse events are not uncommon and should be interpreted with caution. Moreover, these reports emphasize careful patient selection, typically excluding neonates and very low-weight infants.

Emerging multicenter observational data in pediatric robotic surgery (across multiple specialties) have shown that complication rates are comparable to established minimally invasive techniques when performed in experienced centers (23). These findings support the potential for safe integration of robotic thoracic surgery into pediatric practice within structured institutional frameworks, while highlighting the need for further high-quality evidence.

In the comparative cohort studies evaluating RATS and VATS, operative times were consistently longer in the RATS group (15–17). This difference likely reflects docking time and the learning curve associated with robotic systems. The learning curve for robotic thoracic procedures has been studied extensively in adult surgery, where proficiency appears to be achieved after approximately 20–40 cases depending on procedural complexity (24). Pediatric data are less robust, but extrapolation suggests that structured proctoring and team-based robotic training may shorten the adaptation phase (25). Simulation platforms and dry-lab rehearsal have demonstrated measurable improvements in technical efficiency and console time in robotic trainees (26).

Importantly, robotic ergonomics may offer long-term advantages for surgeon sustainability. Musculoskeletal strain is well documented among minimally invasive surgeons, and robotic console systems have been associated with reduced physical fatigue during prolonged procedures (27). While ergonomic benefits do not directly impact perioperative outcomes, they may influence performance consistency and reduce occupational injury over time.

Cost remains a central concern in the adoption of robotic technology. Economic analyses in adult thoracic surgery suggest that robotic procedures incur higher direct hospital costs compared with thoracoscopy, primarily related to disposable instruments and capital expenditure (28). However, some studies indicate that institutional efficiency, case volume, and reduced length of stay may partially offset these differences (28). Pediatric-specific cost analyses are lacking and represent an important area for future research, particularly given the lower-case volumes typical of congenital lung disease.

Despite technological progress, current robotic systems were originally designed for adult anatomy. Port spacing requirements (8 cm preferred, 5–6 cm feasible for pediatrics), instrument diameter (8 mm cannula is standard), and arm geometry can present challenges in small pediatric thoraces (11, 29, 30). The development of smaller-diameter instruments and more compact robotic architectures is underway and may expand applicability to younger infants (30). Preclinical evaluation of single-port robotic systems suggests potential advantages in confined spaces, though depth requirements (10 cm) and internal articulation constraints currently limit routine pediatric use (31).

Long-term pulmonary function and thoracic development after minimally invasive lung resection in children remain critical considerations. Studies evaluating compensatory lung growth after lobectomy have demonstrated favorable remodeling in early childhood (32). Whether robotic approaches influence postoperative pulmonary mechanics differently from thoracoscopic techniques remains unknown. Prospective longitudinal assessment incorporating spirometry, imaging-based volumetry, and quality-of-life instruments will be necessary to clarify potential differences.

The future integration of robotics in pediatric thoracic surgery will likely depend on several synergistic advancements. First, miniaturization of robotic instruments and refinement of arm articulation are essential to expand eligibility to smaller patients. Second, image-guided navigation and three-dimensional reconstruction may complement robotic precision, particularly for anatomical segmentectomy and atypical malformations (33). Third, multicenter collaborative registries are required to generate adequately powered datasets capable of evaluating rare adverse events and long-term outcomes.

## 5 Conclusion

Robotic-assisted thoracic surgery for congenital lung disease in children represents a promising but still maturing technology. Robotic-assisted thoracic surgery offers potential technical advantages, such as improved visualization and instrument articulation; however, these benefits remain largely theoretical in the pediatric population and are not yet supported by robust clinical evidence. Continued technological refinement, structured training programs, prospective multicenter data collection, and cost-effectiveness evaluation are essential to define the precise role of robotics in pediatric thoracic surgery.
